# Tyrosine phosphorylation of the N-Methyl-D-Aspartate receptor 2B subunit in spinal cord contributes to remifentanil-induced postoperative hyperalgesia: the preventive effect of ketamine

**DOI:** 10.1186/1744-8069-5-76

**Published:** 2009-12-30

**Authors:** Xiaoping Gu, Xiaoli Wu, Yue Liu, Songqin Cui, Zhengliang Ma

**Affiliations:** 1 Department of Anesthesiology, Drum Tower Hospital, Medical Department of Nanjing University, Nanjing 210008, Jiangsu Province, China

## Abstract

**Background:**

Experimental and clinical studies showed that intraoperative infusionof remifentanil has been associated with postoperative hyperalgesia. Previous reports suggested that spinal N-methyl-D-aspartate (NMDA) receptors may contribute to the development and maintenance of opioid-induced hyperalgesia. In the present study, we used a rat model of postoperative pain to investigate the role of tyrosine phosphorylation of NMDA receptor 2B (NR2B) subunit in spinal cord in the postoperative hyperalgesia induced by remifentanil and the intervention of pretreatment with ketamine.

**Results:**

Intraoperative infusion of remifentanil (0.04 mg/kg, subcutaneous) significantly enhanced mechanical allodynia and thermal hyperalgesia induced by the plantar incision during the postoperative period (each lasting between 2 h and 48 h), which was attenuated by pretreatment with ketamine (10 mg/kg, subcutaneous). Correlated with the pain behavior changes, immunocytochemical and western blotting experiments in our study revealed that there was a marked increase in NR2B phosphorylation at Tyr1472 in the superficial dorsal horn after intraoperative infusion of remifentanil, which was attenuated by pretreatment with ketamine.

**Conclusions:**

This study provides direct evidence that tyrosine phosphorylation of the NR2B at Tyr1472 in spinal dosal horn contributes to postoperative hyperalgesia induced by remifentanil and supports the potential therapeutic value of ketamine for improving postoperative hyperalgesia induced by remifentanil.

## Background

Numerous experimental and clinical studies have convincingly demonstrated that brief opioid exposure can enhance pain sensitivity that presents as opioid-induced hyperalgesia (OIH) [1]. OIH seem to develop more rapidly and more frequently with the administration of the potent, short-acting opioids such as remifentanil than with longer-acting opioids [2]. Clinically, a relative large dose of intraoperative remifentanil has been shown to trigger postoperative secondary hyperalgesia and to require higher doses of morphine for adequate analgesia [3]. In a healthy human volunteers study, the authors found that a skin area with pre-existing mechanical hyperalgesia was significantly enlarged after a 60- to 90-min remifentanil intravenous infusion [4]. Despite extensive studies in this domain, the possible mechanisms underlying this phenomenon are still unclear.

It is suggested that spinal N-methyl-D-aspartate receptors (NMDARs) dependent central sensitization, a state that dorsal horn excitability is increased and its response to sensory input is facilitated, may be responsible for the opioid-induced hyperalgesia [5]. Because central sensitization is considered to reflect the synapses plasticity in spinal cord, the spinal cord has been the principal focus for studies of mechanisms of hyperalgesia. Electrophysiological studies using slices of rat spinal cord showed that both remifentanil per se and glycine contained in the commercial preparation of remifentanil could elicit rapid and prolonged up-regulation of NMDA receptor function [6,7]. Moreover, Clinical research reporting that NMDA receptor antagonists such as ketamine have the ability to inhibit hyperalgesia induced by remifentanil has also suggested a possible implication of NMDA receptor in the phenomena mentioned above [3].

NMDARs are heteromultimeric complexes that are comprised of at least two types of subunits, the principal subunit NR1 and the modulatory subunit NR2A-D [8]. Particularly crucial are the NMDAR 2B (NR2B) subunit, it has an important function in spinal dorsal horn sensory pathways [9]. Our previous studies have shown that NR2B play an important role in the development of neuropathic pain and cancer pain [10,11]. Protein tyrosine phosphorylation regulates NMDA channel receptor activity. The NR2B receptor has been identified as major tyrosine phosphorylated protein in the postsynaptic density and plays a key role in the signal transduction pathways for NMDA receptor activation [12]. It has been reported that tyrosine phosphorylation of the NR2B subunit plays a role in the induction of long-term potentiation (LTP), a phenomenon related to central sensitization [13,14]. In addition, tyrosine phosphorylation of the NR2B subunit in the rat spinal dorsal horn is closely related to the initiation and development of inflammatory hyperalgesia [15]. These studies suggested an implication of tyrosine phosphorylation of NR2B in the enhancement of synaptic efficacy and thus the development of central sensitization. However, there have been no reports on whether tyrosine phosphorylation of NR2B is associated with the postoperative hyperalgesia induced by remifentanil.

Ketamine, a noncompetitive NMDA receptor antagonist, is a widely used general anesthetic because of its analgesic effects. Although ketamine acts on a variety of receptors, the analgesic effect of ketamine mainly stem from its NMDA-receptor antagonistic properties through prevention of central sensitization [16]. Ketamine inhibits the NMDA receptor by two distinct mechanisms: (1) Ketamine blocks the open channel and thereby reduces channel mean open time, and (2) ketamine decreases the frequency of channel opening by an allosteric mechanism [17]. Therefore it is routinely served as a useful tool to study the role of NMDA receptor in a wide variety of animal and human models. In previous studies, subcutaneous pretreatment with ketamine 10 mg/kg completely prevented the development of long-lasting hyperalgesia after systemic opioid administration in rats [18,19], as well as long-lasting hyperalgesia induced by intrathecal morphine in rats [20].

According to the scenario mentioned above, we hypothesized that tyrosine phosphorylation of the NR2B subunit in spinal cord would contribute to the hyperalgesia induced by remifentanil, which may be prevented by pretreatment with ketamine. In a rat model of postoperative pain, using immunohistochemistry and immunoblotting, we tested this hypothesis through examining tyrosine phosphorylation of NR2B subunit at Tyr1472 in the superficial spinal cord after intraoperative infusion of remifentanil with or without pretreatment of ketamine.

## Methods

### Animals

Experiments were performed on adult male Sprague-Dawley rats (weighing 220-250 g, obtained from the Laboratory Animal Center of Drum Tower Hospital). Animals were housed in groups of four per cage with a 12-h light-12-h dark cycle (lights on at 7:00 AM) at a constant room temperature of 22 ± 2°C. The animals had access to food and water ad libitum. All experiments were approved by our Institutional Animal Care and Use Committee and conform to the guidelines for the use of laboratory animals [21]. Every effort was made to minimize animal suffering and to use the minimum number of animals necessary to obtain valid results.

### Drugs

Remifentanil hydrochloride (batch number: 081101, Ren Fu Co, China), ketamine hydrochloride (batch number: KH080601, Heng Rui Co, China), sevoflurane (batch number: 08100931, Heng Rui Co, China). Ketamine (10 mg/kg) was dissolved in saline (NaCl 0.9%) to a volume of 0.1 ml. Remifentanil (0.04 mg/kg) was dissolved in saline (NaCl 0.9%) to a volume of 0.4 ml. Subcutaneous ketamine injection (10 mg/kg, 0.1 ml) was performed 30 min before plantar incision. Remifentanil (0.04 mg/kg, 0.4 ml) was infused subcutaneously over a period of 30 min using an apparatus pump. The infusion rate was 0.8 ml/h. Control animals received the same volume of saline in identical conditions.

### Surgical procedure

The incisional surgery was based on the procedure described by Brennan et al [22]. Rats were anesthetized with sevoflurane (induction, 3%; surgery, 1%) via a nose mask. The plantar aspect of right hind paw was prepared in a sterile manner with 5% povidone-iodine solution, and the foot was placed through a hole in a sterile drape. A longitudinal 1-cm incision was made through the skin and fascia, starting at 0.5 cm from the edge of the heel and extending toward the toes of the right hind paw. The plantaris muscle was elevated using forceps and incised longitudinally, leaving the muscle origin and insertion intact. After hemostasis with gentle pressure, the skin was closed with two mattress sutures of 5-0 nylon. The wound site was covered with aureomycin ointment.

### Behavioral testing

To evaluate mechanical hyperalgesia, paw withdrawal mechanical thresholds (PWMT) were determined by using calibrated von Frey filaments (2-15 g bending force; Stoelting Co). Rats were placed individually in a cage (22 cm × 12 cm × 12 cm) with a wire mesh bottom (1 cm × 1 cm). Each von Frey filament was applied vertically to an area adjacent to the wound for six seconds while the filament was gently bent. The test was repeated five times with a five-minute test-free period between withdrawal responses. A positive response was defined as complete lifting of the hind paw off the surface of the cage or flinching. The PWMT was determined by the "up-and-down" method and the data were analyzed using the nonparametric method of Dixon, as described by Chaplan et al [23].

To evaluate thermal hyperalgesia, paw withdrawal thermal latency (PWTL) was measured by using testing equipment (BME410A, Institute of Biological Medicine, Academy of Medical Science, China). Rats were placed in a clear plastic chamber (22 cm × 12 cm × 12 cm) with a glass floor (2 mm thick). A radiant heat source was positioned under the glass floor and focused on the plantar surface adjacent to the wound of right hind paw. The withdrawal latency to the thermal stimulation was defined as the time from onset of radiant heat to withdrawal of the rat hind paw. A cutoff time of 25 s was established to prevent tissue damage. There were five trials per rat, taken at 5 min intervals. The mean PWTL equal to the average of the latter three stimuli.

### Experimental Protocol and Groups of Experiments

All rats were anesthetized with sevoflurane (induction, 3%; surgery, 1%) via a nose mask during 30 min. We performed the experiments using five groups of rats (n = 12): group C (rats underwent a sham procedure that consisted of the administration of sevoflurane and the same volume of saline without incision); group I (rats underwent a surgical incision and the same volume of saline); group K (subcutaneous injection of ketamine 30 min before plantar incision); group R (rats underwent a surgical incision and remifentanil was infused subcutaneously at the moment of surgical incision over a period of 30 min); group K+R (subcutaneous injection of ketamine 30 min before plantar incision in remifentanil-treated rats).

After arrival, animals were left to become accustomed to the animal care unit for 5 days. For 2 weeks before the experiments, the animals were weighed daily, handled gently during 5 min, and placed in the test room for 2 h (from 9:00 AM to 11:00 AM), where they were left to become accustomed to the various apparatuses. All experiments were performed by the same investigator in a quiet test room close to the storage room. All experiments began at 9:00 AM and were performed on groups of 12 animals during the light part of the cycle.

For the von Frey and plantar tests, the animals were familiarized with the special conditions of evaluation in the absence of nociceptive stimulation. After the habituation period, baseline responses were obtained just one day before surgery. The experiments (incision + administration of drugs) were performed 1 day later according to the protocol described above. PWMT and PWTL tests were performed at 2 h, 6 h, 24 h, and 48 h after the surgical procedure. The specimens for Immunohistochemical staining and Western blot analysis were collected just after behavioral testings at 48 h.

### Immunohistochemistry

While under deep anesthesia (5% sevoflurane), rats were perfused transcardially with saline, followed by freshly prepared 4% paraformaldehyde in 0.1 M phosphate buffer saline (PBS, pH = 7.4). The lumbosacral spinal cord was dissected out, postfixed in the same fixative at 4°C overnight and then followed by paraffin wax imbedding. Transverse sections (4 μm) were cut through the lumbar L4-5spinal cord by a paraffin slicing machine. Paraffin sections were dewaxed in xylene and rehydrated through graded ethanol dilutions. Endogenous peroxidase activity was inhibited by incubation of slides in 3% hydrogen peroxide solution at room temperature for 30 minutes, followed by three washes 5 minutes in PBS. Next, slides were incubated with 20% normal goat serum for 30 minutes at room temperature and then were diluted with primary antibody (phospho-Tyr1472 NR2B, 1:1000, abcam, Biotechnology, English) at 4°C for 24 h. Sections were then washed 5 minutes in PBS three times, followed by incubation with goat anti-rabbit serum (1:200) for 30 minutes at room temperature followed by three washes 5 minutes in PBS. Antibody localization was detected by addition of the DAB chromogen for 1-5 minutes until staining was visible, followed by washing with PBS/water. Slides were counterstained with Haematoxylin, dehydrated with graded ethanols and covered with gelatin. Slides were observed with an Olympus optic microscope equipped with an Olympus DP11 camera and analysed on-line using Image Pro Plus software (Media Cybernetics, Silver Spring, MD, USA). Each group using six rats and six sections randomly selected from each animal. For each rat, the mean optical density of NR2B tyrosine phosphorylation was obtained by averaging the values from six sections.

### Western Blotting

While under deep anesthesia (5% sevoflurane), the right dorsal horn of the spinal cord L4-L5 segments were removed rapidly and stored in liquid nitrogen. Tissue samples were homogenized in lysis buffer. The homogenate was centrifuged at 13,000 rpm for 10 min at 4°C and supernatant was removed. The protein concentration was determined using Bradford method, a detergent-compatible protein assay with a bovine serum albumin as standard. Samples (70 μg) were separated on SDS-PAGE (6%) and transferred onto a nitrocellulose membrane. The filter membranes were blocked with 5% nonfat milk for 1 h at room temperature andincubated with the primary antibody (phosphor -Tyr 1472 NR2B, 1:500, CST, Biotechnology, USA). The membrane was washed with TBST buffer and incubated for 1 h with the secondary antibody conjugated with horseradish peroxidase (1:5000; Jackson ImmunoResearch, USA) for 1 h at room temperature and visualized in ECl solution for 1 min followed by film exposure for1-10 min. The loading and blotting of equal amount of proteins were verified by reprobing the membrane with antibody against β-actin (1:10,000, Santa Cruz, Biotechnology, USA). The density of specific bands was measured with a computer-assisted imaging analysis system (IPLab software, Scanalytics, Fairfax, VA).

### Statistical Analysis

Data were expressed as the mean ± SD. Repeated measures ANOVA was performed to determine overall differences at each time point in PWMT and PWTL. A one-way ANOVA was used to determine differences in the expression of tyrosine phosphorylation of NR2B across all experiment groups. Post hoc analysis was performed using the LSD test for multiple comparisons in order to determine the differences among experiment groups. The statistical significance criterion was *P *< 0.05.

## Results

### Effects of intraoperative remifentanil infusion on PWMT and PWTL during the postoperative period and the intervention of pretreatment with ketamine

**Figure 1:**
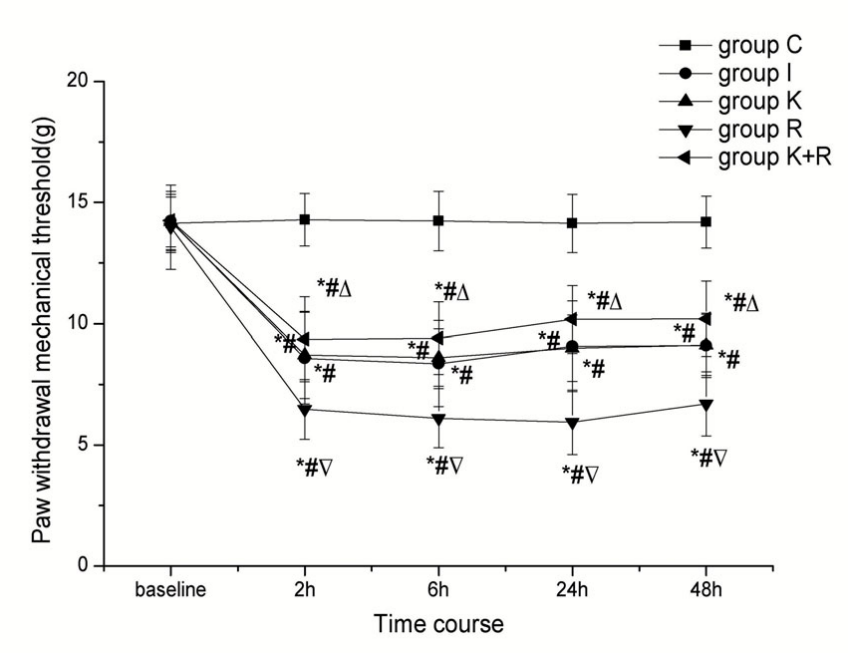
**Effects of remifentanil on PWMT during the postoperative period and the intervention of ketamine**. Ketamine (10 mg/kg, 0.1 ml) or saline was subcutaneously injected 30 min before surgery. Under sevoflurane anesthesia, remifentanil (0.04 mg/kg, 0.4 ml) or saline was subcutaneously infused in the absence or presence of the right hind paw incision during a period of 30 min. PWMT was evaluated at 24 h before incision and at 2 h, 6 h, 24 h and 48 h after surgery. Number of rats per group was twelve. Data are expressed as means ± SD. *P < 0.01 vs baseline, ^# ^*P *< 0.01 vs group C, ^▽^*P *< 0.01 vs group I, ^△^*P *< 0.01 vs group R.

**Figure 2:**
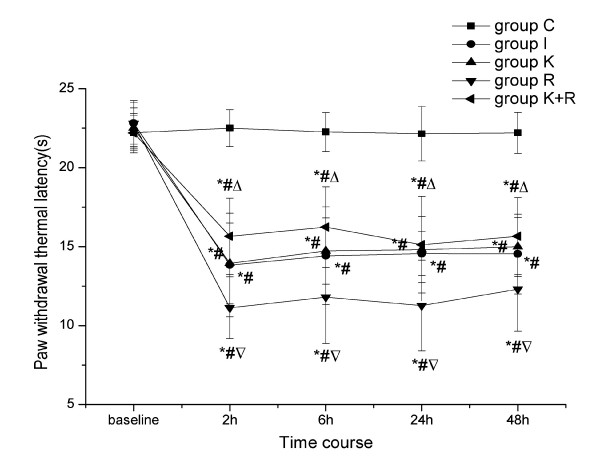
**Effects of remifentanil on PWTL during the postoperative period and the intervention of ketamine**. Ketamine (10 mg/kg, 0.1 ml) or saline was subcutaneously injected 30 min before surgery. Under sevoflurane anesthesia, remifentanil (0.04 mg/kg, 0.4 ml) or saline was subcutaneously infused in the absence or presence of the right hind paw incision during a period of 30 min. PWTL was evaluated at 24 h before incision and at 2 h, 6 h, 24 h and 48 h after surgery. Number of rats per group was twelve. Data are expressed as means ± SD. * *P *< 0.01 vs baseline, ^# ^*P *<0.01 vs group C, ^▽^*P *<0.05 vs group I, ^△^*P *< 0.01 vs group R.

When compared with baseline, the administration of sevoflurane and the subcutaneous infusion of saline for a period of 30 min to rats in the absence of surgery did not produce significant changes of PWMT and PWTL (*P *> 0.05). However, decreases in nociceptive thresholds were observed in other groups from 2 h to 48 h after the surgery. Compared with baseline and group C, the plantar incision induced a decrease in PWMT (*P *< 0.01) and PWTL (*P *< 0.01) in the operated paw during the postoperative period (each lasting between 2 h and 48 h). No statistically significant differences in nociceptive thresholds were observed between group K and group I (*P *> 0.05). Intraoperative infusion of remifentanil significantly enhanced mechanical allodynia and thermal hyperalgesia induced by the plantar incision. This was manifested by a significant decrease in PWMT (*P *< 0.01) and PWTL(*P *< 0.05) below those observed in rats treated with saline in the presence of surgery. Nevertheless, pretreatment with ketamine strongly reduced the enhancement of mechanical allodynia (*P *< 0.01) and thermal hyperalgesia (*P *< 0.01) induced by perioperative remifentanil administration (Figure 1 and Figure 2).

### Immunocytochemical localization and expression of NR2B phosphorylation at Tyr-1472 in spinal cord

**Figure 3:**
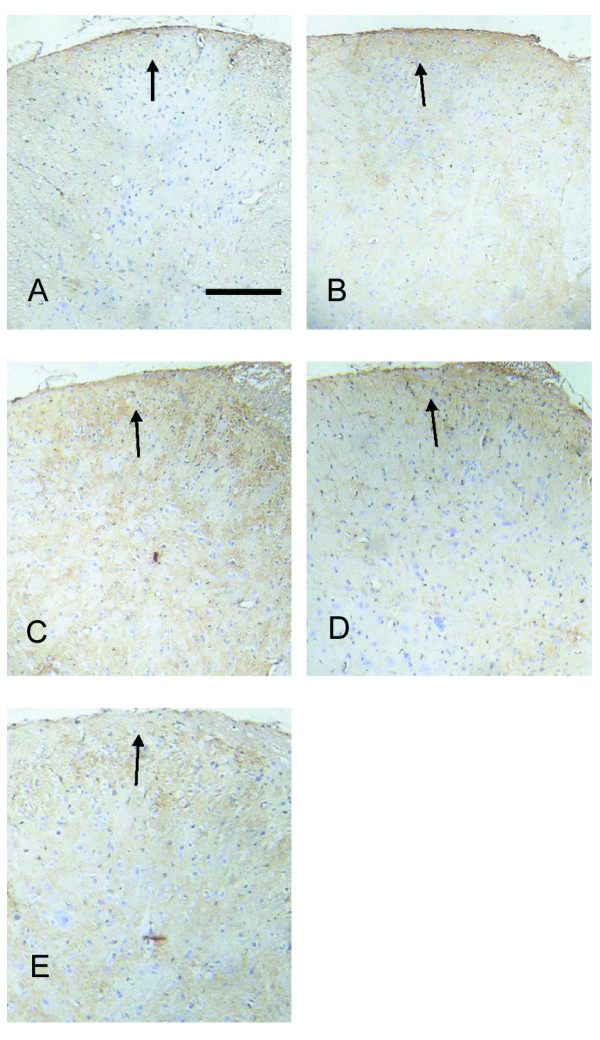
**Typical photomicrographs representing tyrosine phosphorylation of NR2B immunoreactive neurons in the superficial dorsal horn**. The L4-L5 spinal cords for analysis were collected at 48 h after the surgery. In preparations of control group, hardly any tyrosine phosphorylation of NR2B immunoreactive neurons was found in the dorsal horn region (A). In preparations of rats with incision receiving saline or ketamine, moderate tyrosine phosphorylation of NR2B immunoreactive neurons was obtained in the superficial dorsal horn (Laminae I-II) at the L4-L5 spinal cord, ipsilateral to the incision (B and C). The number of tyrosine phosphorylation of NR2B immunoreactive neurons were drastically upregulated in rats receiving infusion of remifentanil (D), which was remarkably inhibited by pretreatment with ketamine (E). Magnification: × 100. Scale bar = 100 μm.

**Figure 4:**
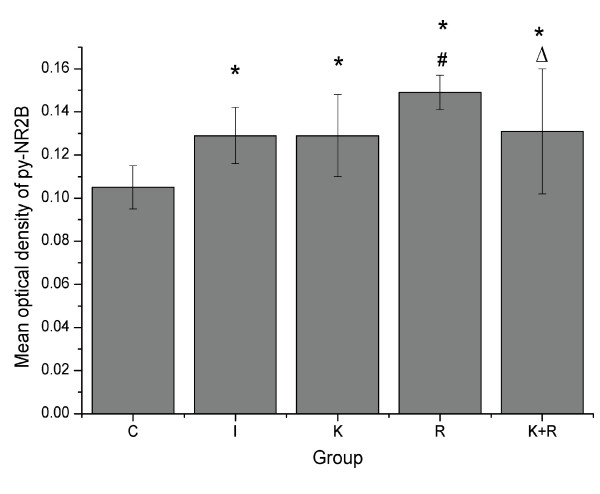
**Immunohistochemical analysis**. The L4-L5 spinal cords for analysis were collected at 48 h after the surgery. The mean optical density of NR2B tyrosine phosphorylation (py-NR2B) in the superficial dorsal horn (Laminae I-II) at the L4-L5 spinal cord are summarized. Data from five groups, each using six rats. For each rat, the mean optical density of NR2B tyrosine phosphorylation was obtained by averaging the values from six sections. Data are expressed as means ± SD. * *P *< 0.01 vs group C, ^# ^*P *< 0.05 vs group I, ^△^*P *< 0.05 vs group R.

To localize and assess the expression of NR2B phosphorylation at Tyr-1472 in the spinal cord during the maintenance of hyperalgesia induced by remifentanil, immunohistochemical studies were performed. Typical photomicrographs showed that the immunohistochemical location of the phosphorylation NR2B subunit was in the superficial dorsal horn (Laminae I-II) at the L4-L5 spinal cord, ipsilateral to the incision (Figure 3). The mean optical density of NR2B tyrosine phosphorylation in the superficial dorsal horn was summarized in Figure 4. The expression of NR2B tyrosine phosphorylation in the superficial dorsal horn was weak in rats receiving sevoflurane and saline without surgery. The expression of NR2B tyrosine phosphorylation in the superficial dorsal horn of the group I, K, R, K+R was increased when compared to group C (*P *< 0.01). No significant change was found between group I and group K(*P *> 0.05). Intraoperative infusion of remifentanil significantly enhanced the expression of NR2B tyrosine phosphorylation in the superficial dorsal horn of spinal cord(*P *< 0.05). Conversely, pretreated ketamine decreased the higher level of NR2B tyrosine phosphorylation in spinal dorsal horn caused by intraoperative infusion of remifentanil (*P *< 0.05).

### Western Blot Analysis

**Figure 5:**
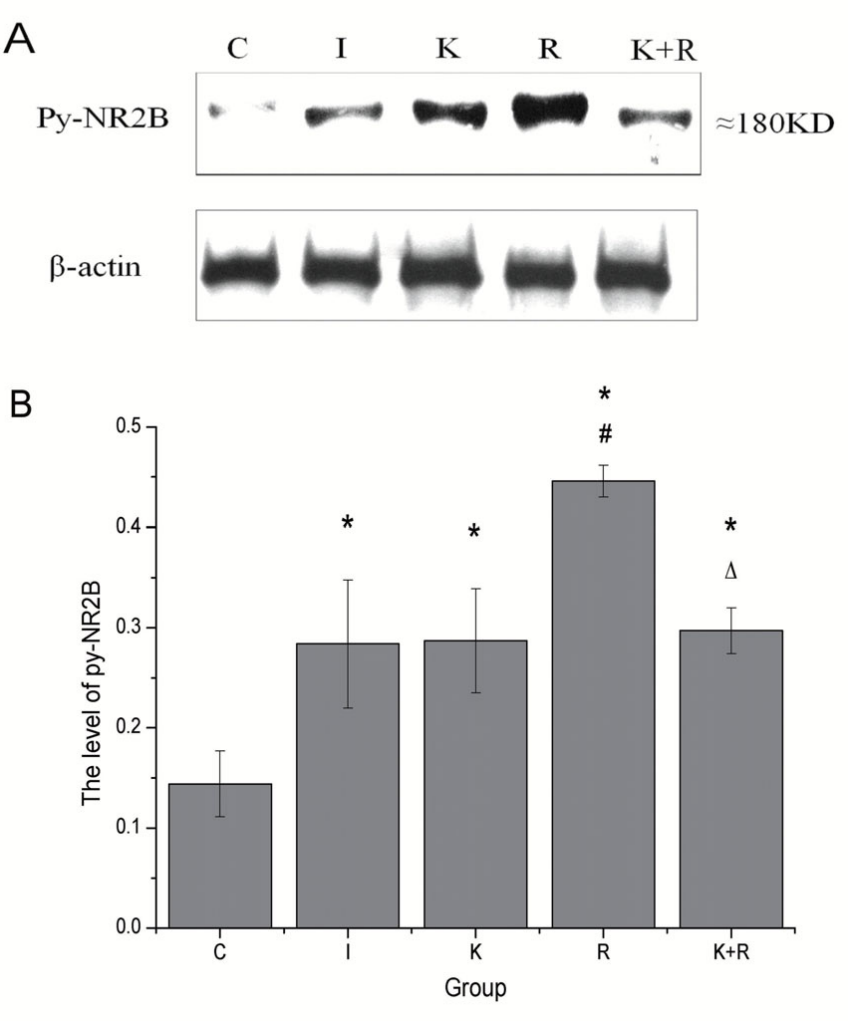
**The expression level of tyrosine phosphorylation of NR2B in superficial spinal cord in each group**. The L4-L5 spinal cords for analysis were collected at 48 h after the surgery. Proteins were extracted from the dorsal half of the L4-L5 spinal cord.(A) Representative western blot for tyrosine phosphorylation of NR2B (py-NR2B) in the superficial dorsal horn at the L4-L5 spinal cord; (B) quantification of NR2B tyrosine phosphorylation in each group. Data from five groups, each using six rats. Data are expressed as means ± SD.* *P *< 0.01 vs group C, ^# ^*P *< 0.01 vs group I, ^△^*P *< 0.01 vs group R.

To quantificate the expression of NR2B phosphorylation at Tyr-1472 in the dorsal horn during the maintenance of hyperalgesia induced by remifentanil, western blot studies were performed. When compared with rats receiving sevoflurane and saline in the absence of surgery, the right hind paw plantar incision increased the level of NR2B tyrosine phosphorylation in the spinal dorsal cord (*P *< 0.01). No significant change was found between group I and group K (*P *> 0.05). The expression of NR2B tyrosine phosphorylation in rats receiving intraoperative remifentanil infusion was significantly upregulated when compared with rats receiving intraoperative saline infusion (*P *< 0.01). Conversely, pretreatment of ketamine decreased the higher level of NR2B tyrosine phosphorylation in spinal dorsal horn caused by remifentanil (*P *< 0.01) (Figure 5).

## Discussion

There are two main findings in this study. The first one is that hyperalgesia induced by remifentanil in a rat model of postoperative pain associated with an enhancement of tyrosine phosphorylation of NR2B in superficial spinal cord. The second finding is that pretreatment with ketamine, via inhibiting tyrosine phosphorylation of NR2B in superficial spinal cord of rats, attenuated exaggerated hyperalgesia induced by remifentanil.

It has been reported that a relatively large dose of intraoperative remifentanil triggers postoperative secondary hyperalgesia in humans. However, it is difficult to discern pain due to tissue damage from hyperalgesia stem from both nociceptive inputs and remifentanil during the postoperative period in clinical studies. So it would be an effective method that using a rat model of postoperative pain to investigate the mechanism of the remifentanil-induced hyperalgesia. In this model, we imitated the administration of general anesthesia in humans which including inhalation of sevoflurane and subcutaneous infusion of remifentanil at constant speed. The doses of remifentanil (0.04 mg/kg) in our study were selected according to literature in rats showing that administration of remifentanil produces a loss of righting reflex that is predictive of clinical anesthesia [24]. In the present study we observed that the infusion of remifentanil during surgery produced rapid and prolonged mechanical allodynia and thermal hyperalgesia during the postoperative period for up to 2 days. The results are consistent with Celerier's study showing that intraoperative subcutaneous infusion of remifentanil in a mice model of postoperative pain enhances the mechanical allodynia and thermal hyperalgesia (each lasting between 2 and 7 days) resulting from surgical incision [25].

Accumulated evidences have indicated that activation of NMDA receptor play a key role in the development and maintenance of central sensitization states underlying the behavioral manifestations of hyperalgesia [26]. Once central sensitization is established, it does not require ongoing peripheral inputs from the injured tissue and then constitute a pathophysiologic mechanism underlying pain hypersensitivity states [27]. Protein phosphorylation is important for the up-regulation of NMDAR function. NR2 is tyrosine phosphorylated at its C-terminal tails by Src-family tyrosine kinases, such as Src, Fyn [28,29]. Although both NR2A and NR2B subunits can be tyrosine phosphorylated in vitro, studies have showed that tyrosine phosphorylation of the NR2B subunits play a key role in the NMDA receptor activation, and contribute to nociceptor activity induced spinal plasticity and the development of central sensitization [30]. The NR2B subunit contains a number of potential tyrosine phosphorylation sites in the cytoplasmic C-terminal tails. Among those tyrosines, Tyr-1472 appears to be the major Fyn-mediated phosphorylation site. Tyr-1472 phosphorylation is important for synaptic plasticity and plays a role in the induction of LTP [31], which shares pharmacology and signal transduction pathways with OIH [32]. It has been reported that tyrosine phosphorylation of the NR2B at Tyr1472 in the spinal dorsal horn contribute to the development of hyperalgesia in inflammation pain model, as well as neuropathic pain model [30,33]. Moreover, suggestion that the mechanisms of central sensitization induced by opiates share some common pathways with those induced by nociceptive inputs [34] led us to speculate that tyrosine phosphorylation of the NR2B subunit at Tyr1472 in spinal cord may be involved in the hyperalgesia induced by remifentanil.

This hypothesis is supported by immunohistochemical and westernblotting experiments in our study showing that remifentanil significantly increased the expression of tyrosine phosphorylation of NR2B in the lamina I-II of the L4-L5 spinal cord. So the activation of NMDARs positively correlates with phosphorylation of NR2B at Y1472 may be a component of underlying mechanisms of hyperalgesia induced by remifentanil. Moreover, because NMDA receptors are activated by the co-agonists glutamate and glycine [35], the role of glycine contained in the commercial preparation of remifentanil cannot be excluded. Our study reports for the first time in an vivo model of hyperalgesia induced by remifentanil that tyrosine phosphorylation of NR2B at Tyr1472 in spinal dorsal horn plays a key role in hyperalgesia induced by remifentanil. Although in our study we cannot confirm the precise mechanisms, we hope our results could provide clue to further explore the possible mechanisms regarding its signaling pathways in the spinal cord after administration of remifentanil.

Of note is our finding that subcutaneous pretreatment with ketamine 10 mg/kg could prevent the development of mechanical allodynia and thermal hyperalgesia induced by remifentanil and the effects are long lasting. Although at present considerable controversy exists regarding the effects of ketamine on the hyperalgesia induced by remifentanil in humans [3,36], our result is consistent with most clinical reports suggesting that ketamine pretreatment could prevent such a hyperalgesia induced by remifentanil. Interestingly, we observed that pretreatment with ketamine 10 mg/kg had no effect on the nociceptive threshold if administered alone, but it prevented the delayed hyperalgesia induced by remifentanil for two days, meaning the preventive effects of this dose of ketamine on hyperalgesia mainly stem from its antihyperalgesia properties, but not antinociceptive effects.

Correlated with the antihyperalgesic and antiallodynic effects of ketamine, in the present study immunocytochemical and western blotting experiment revealed pretreatment with ketamine 10 mg/kg reduced the higher level of NR2B tyrosine phosphorylation in spinal dorsal horn caused by remifentanil. Wilson's study showed that the increased expression of NR2B in the superficial dorsal horn of animal models of neuropathic pain was attenuated following NMDA receptor antagonist pretreatment [37]. Furthermore, there were studies observed that increased NR2B tyrosine phosphorylation after LTP could blocked by MK-801 (an NMDAR channel blocker), suggesting that tyrosine phosphorylation of NR2B is triggered by activation of the NMDAR itself [13,14]. Collectively, these studies in combination with our current data would argue that the antihyperalgesic and antiallodynic effect of ketamine may be linked, at least partially or indirectly, to its ability to inhibit tyrosine phosphorylation of NR2B in the spinal dorsal horn. Additionally, other factors such as blocking voltage-dependent calcium channel [38,39], inhibition of NO synthase [40] and involvement of opiate and monoaminergic neuronal systems [41,42] cannot be excluded at present, which requires additional investigation.

## Conclusions

This study showed that the expression of NR2B tyrosine phosphorylation in the spinal dorsal horn increases markedly during the maintenance of the hyreralgesia induced by remifentanil and this increase can be prevented by pretreatment with ketamine. In summary, the present investigation provides evidence for the significant role of NR2B tyrosine phosphorylation in spinal cord in the maintenance of hyreralgesia induced by remifentanil. In addition, this study supports the potential therapeutic value of ketamine for the prevention of this phenomenon and further suggests that the NR2B-selective antagonist hold promise as novel therapeutics for the control of remifentanil induced hyperalgesia.

## List of abbreviations used

NMDA: N-methyl-D-aspartate; NR2B: N-Methyl-D-Aspartate receptor 2B subunit; Py-NR2B: tyrosine phosphorylation of NR2B; Tyr-1472: tyrosine 1472; LTP: long-term potentiation.

## Competing interests

The authors declare that they have no competing interests.

## Authors' contributions

All authors read and approved the manuscript. XPG and XLW carried out the administration of drugs, surgical procedure, immunohistochemistry and western blots studies and drafted the manuscript; YL and SQC were responsible for pain behavioral tests and statistical analysis; ZLM conceived the idea, designed the study and helped to draft the manuscript for this study.
